# Number of Teeth and Incidence of Hip Fracture in Older Adults Aged ≥75 Years: The OHSAKA Study

**DOI:** 10.2188/jea.JE20240165

**Published:** 2025-07-05

**Authors:** Naoko Otsuki, Tomoaki Mameno, Yuya Kanie, Masahiro Wada, Maki Shinzawa, Kazunori Ikebe, Ryohei Yamamoto

**Affiliations:** 1 Health and Counseling Center, Osaka University, Osaka, Japan; 2 Community-based Integrated Care Science, School of Nursing, Osaka Metropolitan University, Osaka, Japan; 3 Department of Removable Prosthodontics and Gerodontology, Osaka University Graduate School of Dentistry, Osaka, Japan; 4 Department of Orthopaedic Surgery, Osaka University Graduate School of Medicine, Osaka, Japan; 5 Laboratory of Behavioral Health Promotion, Department of Health Promotion Medicine, Osaka University Graduate School of Medicine, Osaka, Japan

**Keywords:** hip fractures, public dental check-ups, tooth loss, older adults, longitudinal study

## Abstract

**Background:**

Several studies reported an association between the number of teeth and the incidence of hip fractures in observational studies, mainly in middle-aged adults. This retrospective cohort study aimed to clarify the association between the number of teeth and the incidence of hip fractures.

**Methods:**

In this retrospective cohort study, a total of 256,772 participants aged 75 years or older who underwent public dental checkups in Japan were evaluated. Exposure in this study was the number of teeth, with a maximum number of 28, excluding third molars. Outcome measures were the incidence of hip fractures needing surgery, using the Japanese procedure codes in medical claims.

**Results:**

A total of 190,998 participants met the inclusion criteria and were available for analysis. Adjusted Fine and Gray models identified a significant association between the number of teeth, including sound, filled, and decayed teeth, and the incidence of hip fractures among women but not for men. The continuous net reclassification improvement of the sound and filled teeth count model increased by 0.078 compared with that of the sound, filled, and decayed teeth count model among women.

**Conclusion:**

The number of sound and filled teeth predicted the risk of hip fractures in women, whereas no association was observed between the number of teeth and hip fractures in men.

## INTRODUCTION

Older adults are prone to fragility fractures such as hip fractures.^1^ Hip fractures remain a global public health concern, with a prevalence of ≥10 million cases per year globally.^2^ The lifetime risk of a hip fracture from age 50 years onward has been estimated at 17% for women and 6% for men,^3^ indicating that hip fractures are common among older adults.^4^ The prevention of hip fractures is highly significant in older adults because those with a hip fracture are at substantial risk of becoming bedridden,^5^ cardiovascular diseases,^6^ strokes,^6^ respiratory diseases,^7^ and mortality.^8^^–^^10^

The main cause of hip fractures in older adults is falls,^1^^,^^11^ which are often triggered by a decline in functional status and poor posture balance.^12^^,^^13^ Previous studies showed that tooth loss was a risk factor for low functional status and poorer postural instability.^14^ Another critical factor of hip fracture is osteoporosis, which might be exacerbated by malnutrition in older persons with impaired chewing function due to a reduced number of teeth.^15^ A cross-sectional study reported that older adults with fewer teeth were at poorer nutritional status.^16^ Several observational studies reported an association between the teeth number and the incidence of hip fractures, which included middle-aged adults with an average age of 53.9^17^ and 61.1^18^ years, who were at low risk of hip fracture. Few study assessed the association between teeth number and hip fracture among older adults.^19^ The clinical impact of teeth number on hip fracture in older adults should be ascertained in a large cohort study.

The present retrospective cohort study aimed to clarify the association between the teeth number and the incidence of hip fractures among 256,772 older adults aged ≥75 years during a median observational period of 38 months. Additionally, we assessed which teeth should be counted for the prediction of hip fracture, sound, filled, or decayed teeth.

## METHODS

### Participants

This longitudinal study was part of the ongoing large cohort study from the Oral Health Screening to Assess Keys of Aging well (OHSAKA) study that aims to identify factors contributing to healthy life expectancy among older adults aged ≥75 years. The eligible participants of this retrospective cohort study were older adults aged ≥75 years who underwent annual dental checkups during the entry period between April 2018 and March 2020 in Osaka Prefecture, Japan. In Japan, most older persons aged ≥75 years belong to the late-stage medical care system for older adults, besides a minority of persons aged 65–74 years with disabilities. A total of 1,100,572 beneficiaries who had an insured period on April 1, 2018, were eligible for this study. Of these beneficiaries, 256,772 participants underwent annual dental checkups in Osaka during the entry period. Those with long-term hospitalization and residents at nursing homes were not eligible for this annual health checkup program. The baseline date was set at the first dental checkup during the entry period. We finally included 190,998 participants aged ≥75 years at the baseline dental checkup after excluding 5,182 (2.0%) participants with unavailable information on long-term care insurance in a single city; 1,850 (0.7%) participants aged <75 years at the baseline dental checkup; 27,503 (10.7%) participants with <6 months of insurance period before the baseline dental checkup; 19,692 (7.7%) participants with missing number of teeth variables at the baseline dental checkup, excluding third molars; 11,340 (4.4%) participants with missing covariates at the baseline dental checkup; and 207 (0.1%) participants with no follow-up period after the baseline dental checkup (eFigure 1).

All insurance and medical claim data were retrieved from the national health insurance database of Japan (Kokuho database [KDB]). All dental checkup data was provided by the Extended Association of Medical Care System for Older Senior Citizens of Osaka Prefecture, Japan. Since April 2018, public dental checkups have been provided annually for older adults in the late-stage medical care system for the older in the latter stage of life.^20^

### Ethical considerations

The study protocol was approved by the ethics committees of the Health and Counseling Center, Osaka University (Approval Number: 2023–2) and Graduate School of Dentistry (Approval Number: R4–M2–1), Osaka University.

### Measurements

#### Exposure

The main exposure of this study was the number of teeth, with a maximum number of 28, excluding third molars. All teeth were diagnosed as sound, filled, or decayed by dentists with participants on a dental chair at their dental clinics. In this study, to assess the dose-dependent association between the number of teeth and the incidence of hip fractures, the distribution of the number of teeth was confirmed, and the categories for the number of teeth were set to 0, 1–5, 6–10, 11–15, 16–20, and 21–28.

#### Covariates

Baseline variables included age,^18^ sex,^17^^,^^18^ body mass index (BMI; body weight [kg]/height^2^ [m^2^]),^18^ self-reported smoking status (never, past, and current smoking),^18^ functional status,^21^ use of drugs,^17^ use of dentures, hospitalization,^7^ kidney replacement therapy,^7^ and medical cost at the baseline dental checkup.^7^ Baseline functional status was ascertained as certification of long-term care (LTC) needs before the baseline dental checkup. In Japan, LTC needs are based on a 74-item questionnaire on activities of daily living (ADL) performance and physicians’ medical diagnoses. The Care Needs Certification Board of the municipal government determines certification of LTC needs and assigns care needs levels, which comprise of seven levels: requiring support 1 and 2 and requiring LTC 1, 2, 3, 4, and 5.^22^^,^^23^ The use of drugs was determined based on the history of one or more prescriptions during the 6 months before the baseline date. The medical claims codes for drugs were converted to Anatomical Therapeutic Chemical Classification System codes using the master data developed by the Japan Pharmaceutical Information Center (Tokyo, Japan). Antidiabetic drugs were defined as A10 code of anatomical therapeutic chemical classification system by World Health Organization^24^; antiplatelet drugs as B01AC (platelet aggregation inhibitors excluding heparin)^25^; beta blockers as C07^26^; calcium channel blockers as C08^24^; renin-angiotensin system (RAS) blockers as C09^24^; cholesterol-lowering drugs as C10AA (3-hydroxy-3-methylglutaryl-coenzyme A reductase inhibitors), C10AX09 (ezetimibe), and C10BA (combinations of various lipid modifying agents)^27^; antidementia drugs as N06DA (anticholinesterases) and N06DX01 (memantine)^26^; and osteoporosis drugs as M05BA (bisphosphonate, romosozumab), G03XC, G03FA, G03FB (raloxifene hydrochloride, estradiol levonorgestrel), H05BA (elcatonin, calcitonin salmon) and H05AA (teriparatide, abaloparatide acetate, denosumab), respectively. Requiring kidney replacement therapy, such as hemodialysis, peritoneal dialysis, or kidney transplantation, was determined based on the history of one or more medical claims within 6 months before the baseline dental checkup. Other factors, such as hospitalization, kidney replacement therapy, and medical costs, were based on the medical claims within 6 months before the baseline date. Those with hospitalization had a history of at least one episode of this. The medical costs (<10,000, 10,000–49,999, 50,000–99,999, 100,000–199,999, 200,000–299,999, and ≥300,000 yen) were calculated using medical claims on medical procedures, prescriptions, Diagnosis Procedure Combination,^28^ and fee-for-service payment.^28^

#### Outcome

The outcome measures of this study were the incidence of hip fractures needing surgery, using the Japanese procedure codes in medical claims. The competing event was death before the incidence of hip fracture, as verified by the reason for the loss of insurance eligibility in the KDB. The observational period was designated as the number of months from the baseline month to the incidence of hip fracture, death, loss of insurance eligibility, or March 2022, whichever came first. If hip fracture and death occurred in the same month, the incidence of hip fracture was treated as the outcome event before death.

### Statistical analyses

Continuous variables were expressed as median (interquartile range) and categorical variables were expressed as numbers (proportions). Competing risk regression analyses were performed using Fine and Gray proportional sub-distribution hazards models with death as a competing risk event.^29^ The cumulative probability of incidence of hip fracture was estimated using the cumulative incidence function estimator and compared with a weighted log-rank test.^30^^,^^31^ The association between the number of teeth and the incidence of hip fracture was assessed using unadjusted and adjusted Fine and Gray proportional sub-distribution hazard models. Sub-distribution hazard ratios (SHRs) with 95% confidence intervals (CIs) for the six categories of each tooth count method were calculated in unadjusted and multivariable-adjusted models including the age of participants, BMI, LTC needs levels, use of dentures, self-reported smoking status, use of beta-blockers, calcium channel blockers, and RAS blockers and antidiabetic, antiplatelet, cholesterol-lowering, antidementia drugs, and osteoporosis drugs, requiring kidney replacement therapy, hospitalization, and medical cost as covariates.

As sensitivity analyses, first, we assessed the association between the number of teeth (0, 1–5, 6–10, 11–15, 16–20 vs 21–28) and the incidence of hip fractures using inverse probability of treatment weighting (IPTW) to control for the potential confounding factors: age (years); LTC needs level (none, requiring support 1 and 2 and requiring LTC 1, 2, 3, 4, and 5); BMI (<18.5, 18.5–24.9, 25.0–29.9, and ≥30 kg/m^2^); smoking (never, past, and current smoking); use of antidiabetic drugs, antiplatelet drugs, beta-blockers, calcium channel blockers, RAS blockers, cholesterol-lowering drugs, antidementia drugs, and osteoporosis drugs; kidney replacement therapy; hospitalization; and 6-month medical costs (<10,000, 10,000–49,999, 50,000–99,999, 100,000–199,999, 200,000–299,999, and ≥300,000 yen). Second, to control differences in the length of the observational period among six categories of the teeth number, we censored the observational period at 24 and 36 months. Third, the association between teeth number and hip fracture was assessed in participants with use of denture, which was very common among participants with low teeth number.

To evaluate appropriate methods for counting teeth in predicting the risk of hip fractures, we calculated the net reclassification improvement (NRI) using the sound teeth and the sound + filled teeth models, with the sound + filled + decayed teeth count model as the reference.^32^^,^^33^

All data were analyzed using R software, version 4.2.1 (R Foundation for Statistical Computing, Vienna, Austria). All statistical tests were two-sided, and a *P* < 0.05 was considered significant.

## RESULTS

Of 1,100,572 beneficiaries of the late-stage medical care system for the older adults in Osaka Prefecture in April 2018, 256,772 (23.3%) participants underwent annual dental checkups during the entry period between April 2018 and March 2020. After excluding 65,744 (6.0%) participants, the present study finally included 190,998 (17.4%) participants with the baseline variables and the insured period after the baseline dental checkup date (eFigure 1). Compared with 190,998 included participants (81,158 men and 109,840 women), 31,239 excluded participants (12,389 men and 18,850 women) with missing number of teeth variables (*n* = 19,692), missing covariates (*n* = 11,340), and no insured period after the baseline checkups, had the higher prevalence of LTC certification, use of denture, and using dementia drugs (eTable 1 and eTable 2).

The baseline characteristics of 81,158 men and 109,840 women stratified by six categories of the number of the sound + filled + decayed teeth are described in Table 1 and Table 2, respectively. Participants with the larger teeth number were younger and had a lower prevalence of LTC needs, use of antidiabetic, calcium-channel blockers, RAS blockers, antiplatelet, and antidementia drugs, and rate of hospitalization than those with the lower teeth number. Most participants with 15 or fewer teeth were denture wearers (95.3% of men and 95.2% of women).

**Table 1.** Clinical characteristics of 81,158 men stratified by number of sound + filled + decayed teeth

		Number of sound + filled + decayed teeth					

		0	1–5	6–10	11–15	16–20	21–28
Number		4,636	5,931	7,809	8,524	12,311	41,947
Age, years		81 (78–85)	81 (77–84)	80 (77–84)	80 (77–83)	79 (77–83)	78 (76–82)
Long-term care needs levels, *n* (%)							
None		2,275 (49.1)	3,253 (54.8)	4,399 (56.3)	5,067 (59.4)	7,820 (63.5)	29,299 (69.8)
Requiring support	1	998 (21.5)	1,188 (20.0)	1,486 (19.0)	1,448 (17.0)	1,958 (15.9)	5,464 (13.0)
	2	440 (9.5)	465 (7.8)	619 (7.9)	629 (7.4)	798 (6.5)	2,201 (5.2)
Requiring long-term care	1	451 (9.7)	503 (8.5)	623 (8.0)	677 (7.9)	901 (7.3)	2,517 (6.0)
	2	217 (4.7)	205 (3.5)	303 (3.9)	268 (3.1)	352 (2.9)	1,052 (2.5)
	3	102 (2.2)	121 (2.0)	158 (2.0)	167 (2.0)	208 (1.7)	540 (1.3)
	4	97 (2.1)	120 (2.0)	133 (1.7)	153 (1.8)	164 (1.3)	532 (1.3)
	5	56 (1.2)	76 (1.3)	88 (1.1)	115 (1.3)	110 (0.9)	342 (0.8)
Body mass index, *n* (%)							
<18.5 kg/m^2^		395 (8.5)	447 (7.5)	594 (7.6)	510 (6.0)	653 (5.3)	2,066 (4.9)
18.5–24.9 kg/m^2^		3,266 (70.4)	4,278 (72.1)	5,594 (71.6)	6,115 (71.7)	8,861 (72.0)	31,032 (74.0)
25.0–29.9 kg/m^2^		896 (19.3)	1,099 (18.5)	1,488 (19.1)	1,742 (20.4)	2,577 (20.9)	8,185 (19.5)
≥30 kg/m^2^		79 (1.7)	107 (1.8)	133 (1.7)	157 (1.8)	220 (1.8)	664 (1.6)
Smoking, *n* (%)							
Never		1,001 (21.6)	1,337 (22.5)	1,842 (23.6)	2,179 (25.6)	3,315 (26.9)	14,060 (33.5)
Past		2,833 (61.1)	3,613 (60.9)	4,752 (60.9)	5,181 (60.8)	7,482 (60.8)	24,010 (57.2)
Current		802 (17.3)	981 (16.5)	1,215 (15.6)	1,164 (13.7)	1,514 (12.3)	3,877 (9.2)
Use of dentures, *n* (%)		4,520 (97.5)	5,754 (97.0)	7,492 (95.9)	7,878 (92.4)	9,964 (80.9)	11,539 (27.5)
Antidiabetic drugs, *n* (%)		1,078 (23.3)	1,243 (21.0)	1,656 (21.2)	1,807 (21.2)	2,516 (20.4)	7,314 (17.4)
Antiplatelet drugs, *n* (%)		1,627 (35.1)	1,985 (33.5)	2,594 (33.2)	2,740 (32.1)	3,840 (31.2)	12,213 (29.1)
Beta-blockers, *n* (%)		683 (14.7)	803 (13.5)	1,152 (14.8)	1,175 (13.8)	1,687 (13.7)	5,498 (13.1)
Calcium channel blockers, *n* (%)		1,907 (41.1)	2,474 (41.7)	3,285 (42.1)	3,544 (41.6)	4,972 (40.4)	16,086 (38.3)
RAS blockers, *n* (%)		1,847 (39.8)	2,389 (40.3)	3,122 (40.0)	3,443 (40.4)	4,931 (40.1)	15,927 (38.0)
Cholesterol-lowering drugs, *n* (%)		1,426 (30.8)	1,850 (31.2)	2,325 (29.8)	2,455 (28.8)	3,676 (29.9)	12,751 (30.4)
Antidementia drugs, *n* (%)		255 (5.5)	293 (4.9)	337 (4.3)	381 (4.5)	422 (3.4)	1,318 (3.1)
Osteoporosis drugs, *n* (%)		283 (6.1)	332 (5.6)	423 (5.4)	384 (4.5)	619 (5.0)	1,861 (4.4)
Kidney replacement therapy, *n* (%)		30 (0.6)	49 (0.8)	55 (0.7)	72 (0.8)	76 (0.6)	237 (0.6)
Hospitalization, *n* (%)		662 (14.3)	820 (13.8)	1,002 (12.8)	1,059 (12.4)	1,499 (12.2)	4,667 (11.1)
Medical costs for 6 months, *n* (%)							
<10,000, yen		234 (5.0)	320 (5.4)	346 (4.4)	439 (5.2)	514 (4.2)	1,858 (4.4)
10,000–49,999		313 (6.8)	436 (7.4)	597 (7.6)	657 (7.7)	1,026 (8.3)	3,614 (8.6)
50,000–99,999		614 (13.2)	847 (14.3)	1,111 (14.2)	1,277 (15.0)	1,880 (15.3)	6,939 (16.5)
100,000–199,999		1,265 (27.3)	1,604 (27.0)	2,146 (27.5)	2,440 (28.6)	3,451 (28.0)	12,012 (28.6)
200,000–299,999		759 (16.4)	964 (16.3)	1,327 (17.0)	1,400 (16.4)	2,064 (16.8)	6,837 (16.3)
≥300,000		1,451 (31.3)	1,760 (29.7)	2,282 (29.2)	2,311 (27.1)	3,376 (27.4)	10,687 (25.5)
Observational period, months		36 (27–43)	37 (29–44)	38 (30–44)	39 (32–44)	39 (32–44)	40 (32–44)
Incidence of fracture, *n* (%)		113 (2.4)	120 (2.0)	119 (1.5)	129 (1.5)	167 (1.4)	415 (1.0)
All-cause mortality, *n* (%)		913 (19.7)	960 (16.2)	1,193 (15.3)	1,200 (14.1)	1,417 (11.5)	3,688 (8.8)

RAS, renin-angiotensin system.

Data are presented as median (interquartile range) or *n* (%).

**Table 2.** Clinical characteristics of 109,840 women stratified by number of sound + filled + decayed teeth

		Number of sound + filled + decayed teeth					

		0	1–5	6–10	11–15	16–20	21–28
Number		4,662	6,894	11,111	12,369	18,155	56,649
Age, years		83 (79–87)	82 (78–86)	81 (78–85)	80 (77–84)	79 (77–83)	78 (76–82)
Long-term care needs levels, *n* (%)							
None		1,621 (34.8)	2,800 (40.6)	4,859 (43.7)	5,907 (47.8)	9,471 (52.2)	33,135 (58.5)
Requiring support	1	1,451 (31.1)	1,950 (28.3)	3,098 (27.9)	3,245 (26.2)	4,479 (24.7)	12,302 (21.7)
	2	613 (13.1)	881 (12.8)	1,286 (11.6)	1,292 (10.4)	1,838 (10.1)	4,898 (8.6)
Requiring long-term care	1	554 (11.9)	703 (10.2)	1,081 (9.7)	1,136 (9.2)	1,388 (7.6)	3,752 (6.6)
	2	208 (4.5)	260 (3.8)	369 (3.3)	363 (2.9)	472 (2.6)	1,270 (2.2)
	3	99 (2.1)	136 (2.0)	172 (1.5)	191 (1.5)	252 (1.4)	583 (1.0)
	4	70 (1.5)	110 (1.6)	177 (1.6)	172 (1.4)	192 (1.1)	510 (0.9)
	5	46 (1.0)	54 (0.8)	69 (0.6)	63 (0.5)	63 (0.3)	199 (0.4)
Body mass index, *n* (%)							
<18.5 kg/m^2^		697 (15.0)	847 (12.3)	1,319 (11.9)	1,375 (11.1)	1,808 (10.0)	5,961 (10.5)
18.5–24.9 kg/m^2^		3,039 (65.2)	4,677 (67.8)	7,571 (68.1)	8,624 (69.7)	12,726 (70.1)	40,965 (72.3)
25.0–29.9 kg/m^2^		775 (16.6)	1,165 (16.9)	1,892 (17.0)	2,043 (16.5)	3,113 (17.1)	8,496 (15.0)
≥30 kg/m^2^		151 (3.2)	205 (3.0)	329 (3.0)	327 (2.6)	508 (2.8)	1,227 (2.2)
Smoking, *n* (%)							
Never		4,032 (86.5)	5,943 (86.2)	9,567 (86.1)	10,913 (88.2)	16,215 (89.3)	52,451 (92.6)
Past		394 (8.5)	557 (8.1)	924 (8.3)	878 (7.1)	1,187 (6.5)	2,622 (4.6)
Current		236 (5.1)	394 (5.7)	620 (5.6)	578 (4.7)	753 (4.1)	1,576 (2.8)
Use of dentures, *n* (%)		4,531 (97.2)	6,698 (97.2)	10,651 (95.9)	11,458 (92.6)	14,566 (80.2)	14,816 (26.2)
Antidiabetic drugs, *n* (%)		715 (15.3)	972 (14.1)	1,470 (13.2)	1,536 (12.4)	2,197 (12.1)	5,748 (10.1)
Antiplatelet drugs, *n* (%)		1,335 (28.6)	1,854 (26.9)	2,863 (25.8)	3,090 (25.0)	4,422 (24.4)	12,686 (22.4)
Beta-blockers, *n* (%)		503 (10.8)	803 (11.6)	1,257 (11.3)	1,344 (10.9)	1,862 (10.3)	5,462 (9.6)
Calcium channel blockers, *n* (%)		2,080 (44.6)	3,058 (44.4)	4,890 (44.0)	5,306 (42.9)	7,560 (41.6)	21,391 (37.8)
RAS blockers, *n* (%)		1,889 (40.5)	2,710 (39.3)	4,345 (39.1)	4,693 (37.9)	6,618 (36.5)	18,619 (32.9)
Cholesterol-lowering drugs, *n* (%)		1,872 (40.2)	2,723 (39.5)	4,519 (40.7)	5,110 (41.3)	7,702 (42.4)	24,554 (43.3)
Antidementia drugs, *n* (%)		379 (8.1)	405 (5.9)	642 (5.8)	592 (4.8)	806 (4.4)	1,983 (3.5)
Osteoporosis drugs, *n* (%)		1,747 (37.5)	2,558 (37.1)	3,878 (34.9)	4,315 (34.9)	6,283 (34.6)	19,898 (35.1)
Kidney replacement therapy, *n* (%)		22 (0.5)	22 (0.3)	37 (0.3)	34 (0.3)	38 (0.2)	122 (0.2)
Hospitalization, *n* (%)		511 (11.0)	676 (9.8)	1,106 (10.0)	1,093 (8.8)	1,526 (8.4)	4,468 (7.9)
Medical costs for 6 months, *n* (%)							
<10,000, yen		157 (3.4)	250 (3.6)	412 (3.7)	453 (3.7)	653 (3.6)	2,056 (3.6)
10,000–49,999		273 (5.9)	448 (6.5)	703 (6.3)	804 (6.5)	1,319 (7.3)	4,493 (7.9)
50,000–99,999		615 (13.2)	981 (14.2)	1,617 (14.6)	1,880 (15.2)	2,795 (15.4)	9,114 (16.1)
100,000–199,999		1,370 (29.4)	2,159 (31.3)	3,421 (30.8)	3,778 (30.5)	5,604 (30.9)	17,804 (31.4)
200,000–299,999		872 (18.7)	1,219 (17.7)	2,024 (18.2)	2,311 (18.7)	3,367 (18.5)	10,135 (17.9)
≥300,000		1,375 (29.5)	1,837 (26.6)	2,934 (26.4)	3,143 (25.4)	4,417 (24.3)	13,047 (23.0)
Observational period, months		36 (29–43)	38 (30–44)	39 (32–44)	39 (32–44)	40 (32–44)	40 (32–44)
Incidence of fracture, *n* (%)		288 (6.2)	324 (4.7)	475 (4.3)	477 (3.9)	614 (3.4)	1,374 (2.4)
All-cause mortality, *n* (%)		536 (11.5)	568 (8.2)	769 (6.9)	743 (6.0)	931 (5.1)	2,232 (3.9)

RAS, renin-angiotensin system.

Data are presented as median (interquartile range) or *n* (%).

**Figure 1.**
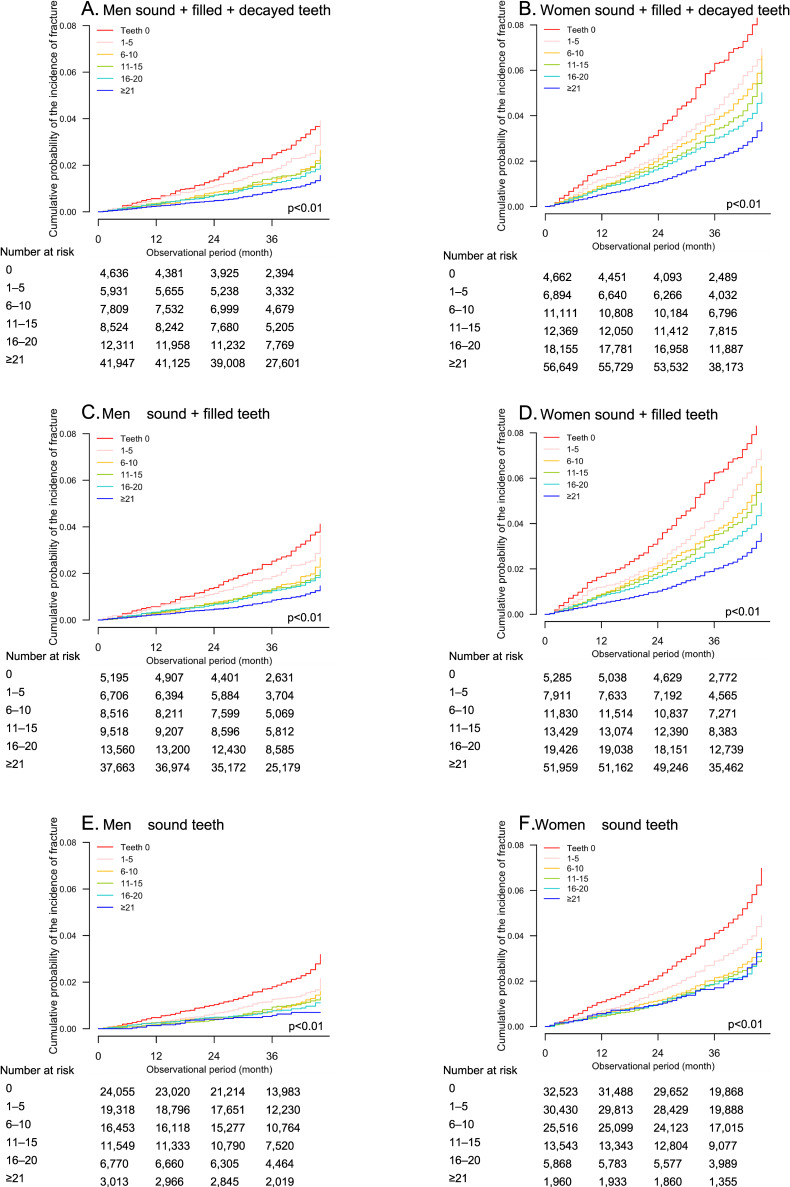
Cumulative probabilities of the incidence of hip fracture in 81,158 men (**A**, **C**, and **D**) and 109,840 women (**B**, **D**, and **F**) stratified by the number of the sound + filled + decayed teeth (**A**, **B**), the sound + filled teeth (**C**, **D**), the sound teeth (**E**, **F**).

After the baseline dental checkups, the participants were followed during the median observational period of 38 (interquartile range, 31–44) months. The participants with lower teeth number had shorter observational period than those with higher teeth number in both men (Table 1) and women (Table 2). The incidence of hip fracture was observed in 1,063 (1.3%) men (Table 1) and 3,552 (3.2%) women (Table 2). The number of sound + filled + decayed teeth was associated with the cumulative probability of the incidence of hip fracture in a dose-dependent fashion among both men (Figure 1A) and women (Figure 1B). A multivariable-adjusted model showed that women with sound + filled + decayed teeth ≤ 20 were at a significantly higher risk for hip fractures than those with 21–28 (adjusted SHRs of 0, 1–5, 6–10, 11–15, 16–20, and 21–28 teeth: 1.28; 95% CI, 1.12–1.47, 1.18; 95% CI, 1.03–1.35, 1.16; 95% CI, 1.03–1.31, 1.18; 95% CI, 1.05–1.31, 1.20; 95% CI, 1.08–1.33, and 1.00 [reference], respectively) (Table 3). In contrast, in men, the number of sound + filled + decayed teeth was not associated with the incidence of hip fracture (1.26; 95% CI, 0.96–1.69, 1.25; 95% CI, 0.98–1.60, 1.03; 95% CI, 0.80–1.31, 1.10; 95% CI, 0.88–1.39, 1.15; 95% CI, 0.91–1.45, and 1.00 [reference], respectively) (Table 3).

**Table 3.** Association of number of teeth and incidence of hip fracture (81,158 men and 109,840 women)

		Number of teeth					

		0	1–5	6–10	11–15	16–20	21–28
Men	Unadjusted SHR (95% CI)						
	S+F+D	2.54 (2.01–3.21)	2.09 (1.71–2.54)	1.54 (1.23–1.92)	1.54 (1.27–1.87)	1.38 (1.14–1.67)	1.00 (reference)
	S+F	2.75 (2.18–3.46)	2.23 (1.84–2.70)	1.59 (1.28–1.97)	1.51 (1.23–1.84)	1.44 (1.21–1.72)	1.00 (reference)
	S	3.10 (1.92–5.01)	2.07 (1.27–3.37)	1.52 (0.91–2.25)	1.45 (0.88–2.39)	1.32 (0.77–2.27)	1.00 (reference)
	Adjusted SHR (95% CI)^*^						
	S+F+D	1.26 (0.95–1.69)	1.25 (0.98–1.60)	1.03 (0.80–1.31)	1.10 (0.88–1.39)	1.15 (0.91–1.45)	1.00 (reference)
	S+F	1.30 (0.97–1.74)	1.23 (0.97–1.57)	1.03 (0.81–1.31)	1.08 (0.84–1.37)	1.13 (0.89–1.43)	1.00 (reference)
	S	1.86 (1.05–3.29)	1.71 (0.97–3.02)	1.38 (0.75–2.54)	1.53 (0.87–2.69)	1.56 (0.82–2.97)	1.00 (reference)

Women	Unadjusted SHR (95% CI)						
	S+F+D	2.69 (2.39–3.03)	2.00 (1.77–2.25)	1.79 (1.61–2.00)	1.60 (1.45–1.76)	1.42 (1.30–1.55)	1.00 (reference)
	S+F	2.77 (2.45–3.15)	2.16 (1.90–2.45)	1.82 (1.63–2.04)	1.68 (1.52–1.86)	1.44 (1.31–1.58)	1.00 (reference)
	S	2.19 (1.64–2.92)	1.54 (1.15–2.05)	1.16 (0.87–1.55)	1.02 (0.75–1.39)	1.02 (0.73–1.42)	1.00 (reference)
	Adjusted SHR (95% CI)^*^						
	S+F+D	1.28 (1.12–1.47)	1.18 (1.03–1.35)	1.16 (1.03–1.31)	1.18 (1.05–1.31)	1.20 (1.08–1.33)	1.00 (reference)
	S+F	1.25 (1.09–1.44)	1.23 (1.07–1.41)	1.14 (1.00–1.30)	1.18 (1.05–1.32)	1.14 (1.02–1.28)	1.00 (reference)
	S	1.28 (0.91–1.80)	1.26 (0.90–1.76)	1.08 (0.77–1.50)	1.10 (0.77–1.57)	1.09 (0.75–1.59)	1.00 (reference)

CI, confidence interval; S+F+D, sound + filled + decayed teeth; S+F, sound + filled teeth; S, sound teeth; SHR, sub-distribution hazard ratio.

^*^Adjusting for age (years); long-term care needs level (none, requiring support 1 and 2 and requiring long-term care 1, 2, 3, 4, and 5); body mass index (<18.5, 18.5–24.9, 25.0–29.9, and ≥30 kg/m^2^); smoking (never, past, and current smoking); use of antidiabetic drugs, antiplatelet drugs, beta-blockers, calcium channel blockers, RAS blockers, cholesterol-lowering drugs, antidementia drugs, and osteoporosis drugs; kidney replacement therapy; hospitalization; and 6-month medical costs (<10,000, 10,000–49,999, 50,000–99,999, 100,000–199,999, 200,000–299,999, and ≥300,000 yen).

Several sensitivity analyses confirmed the association between the number of sound + filled + decayed teeth and the incidence of hip fracture in women. First, the IPTW method showed the association between the number of sound + filled + decayed teeth and the incidence of hip fractures in women (eTable 3). This IPTW method also showed that men with ≤5 teeth were significantly at higher risk for hip fracture than those with 21–28 of sound + filled + decayed teeth (adjusted SHRs of 0, 1–5, and 21–28 teeth: 1.81; 95% CI, 1.18–2.79, 1.96; 95% CI, 1.27–3.04, and 1.00 [reference], respectively), incompatible with the main result. Second, to control differences in the length of the observation period among six categories of sound + filled + decayed teeth, we censored the observation period at 24 and 36 months (eTable 4 and eTable 5, respectively). These findings were similar to those of the main analysis, although several teeth categories were not significantly associated with the incidence of hip fracture, probably because of lower statistical power due to the lower number of the incidence of hip fracture than the main analyses. Third, because use of denture was very common among those with 15 or fewer teeth (95.3% of men and 95.2% of women), the association between teeth number and the incidence of hip fracture was assessed among 47,147 men and 62,720 women with use of denture, confirming the association between the teeth number and the incidence of hip fracture among women, not men (eTable 6).

To compare prediction of the incidence of hip fracture among different methods of counting teeth, we assessed the association of the number of sound + filled teeth and that of sound teeth with the incidence of hip fracture. The association of the number of sound + filled teeth with the incidence of hip fracture was very similar to that of the number of sound + filled + decayed teeth among both men and women (Table 3 and eTable 3). In contrast, the number of sound teeth was not associated with the incidence of hip fracture among women. Among men, those with ≤5 sound teeth were significantly at higher risk for hip fracture than those with 21–28 sound teeth. NRIs was calculated to identify the most effective method of teeth counting for prediction of hip fracture (Table 4). Among women, the sound + filled model predicted hip fracture more accurately than sound + filled + decayed model (NRI for sound + filled + decayed teeth model [reference] vs sound + filled teeth model: 0.078; 95% CI, 0.032–0.107), while the prediction of hip fracture were comparable between the sound + filled + decayed model and the sound model, suggesting that the number of sound + filled teeth predicted the incidence of hip fracture most accurately. In contrast, among men, the sound + filled teeth model and the sound teeth model were comparable with the sound + filled + decayed model.

**Table 4.** Predictive method of counting teeth by reclassification

	Net reclassification improvement (95% confidence interval)	

	Men	Women
S+F+D^*^ vs S+F	0.047 (−0.078–0.132)	0.078 (0.032–0.107)
S+F+D^*^ vs S	−0.038 (−0.160–0.120)	−0.001 (−0.090–0.088)

S+F+D, sound + filled + decayed teeth; S+F, sound + filled teeth; S, sound teeth.

^*^Reference.

Adjusting for age (years), long-term care needs level, body mass index (kg/m^2^), use of antidiabetic drugs, antiplatelet drugs, beta-blockers, calcium channel blockers, RAS blockers, cholesterol-lowering drugs, antidementia drugs, and osteoporosis drugs, kidney replacement therapy, hospitalization, and 6-month medical costs.

## DISCUSSION

This retrospective cohort study, including 190,998 older adults aged ≥75 years, clarified that women with <21 of sound + filled + decayed teeth were at a significantly higher risk of hip fractures than those with ≥21. Among men, the association between the teeth number and the incidence of hip fracture was not conclusive, probably because the low incidence of hip fracture in men led to underpowered analysis. The number of sound + filled teeth predicted hip fracture more accurately than that of sound + filled + decayed teeth in women.

Few studies have reported the association between the teeth number and the incidence of hip fractures. A cross-sectional study including 15,198 adults in the United States showed that those with 28 natural teeth had a significantly lower prevalence of self-reported hip fracture history than those with ≤27.^17^ Another cohort study including 9,992 men in Japan reported that the number of tooth loss was significantly associated with the incidence of hip fractures.^18^ These studies did not compare the predictive power of tooth conditions (ie, sound, filled, and decayed teeth). In this large retrospective cohort study, a detailed classification of teeth conditions enabled us to identify the most effective teeth-counting model to predict the incidence of hip fractures.

Osteoporosis may play an important role in the association between tooth loss and the incidence of hip fractures. Previous studies reported that tooth loss was associated with osteoporosis,^34^^,^^35^ especially in women.^34^ In postmenopausal women, estrogen deficiency causes progressive osteoporosis, a critical factor in hip fractures.^36^ The lifetime incidence of fragility fractures due to osteoporosis has been reported to be 13% in men and 40% in women.^37^ In women, in addition to the progressive osteoporosis due to estrogen deficiency, a smaller skeletal structure and poorer bending strength compared to men are thought to be factors that increase the risk of hip fractures.^36^ In this study, the association between the teeth number and the incidence of hip fractures in women might be confounded by osteoporosis. These findings suggest that screening for osteoporosis might potentially prevent hip fractures in older women with tooth loss.

Other factors supporting the association between tooth loss and hip fracture could include poor body balance due to the lack of occlusal contact. Several studies have reported that the teeth number or occlusal contact in the molar region affected body balance and gravity fluctuation.^14^^,^^38^ Tooth loss or reduced occlusal contact with natural teeth might increase the risk of falling and subsequent hip fractures due to impaired body balance.^14^^,^^37^ Maintaining more teeth potentially preserves occlusal function, which may help prevent hip fractures through the resulting stability in posture balance.^14^^,^^39^ To maintain stability in posture balance during old age, oral care focused on preserving the number of teeth from a young age is crucial.

This study had several limitations. First, because the previous history of hip fractures before the baseline date was unavailable in this study, both first-time and recurrent hip fractures were defined as the outcome. A previous study reported a 7% incidence of contralateral hip fractures among patients with hip fractures.^40^ To clarify the association between the teeth number and the incidence of hip fractures precisely, it is desirable to assess the associations between the teeth number and first-time and recurrent fractures separately. Second, the definition of hip fracture incidence in this study was based on the requirement for surgical treatments according to the medical claims. In older adults, hip fractures are commonly treated conservatively,^41^ with nonsurgical treatments being opted for. The definition of hip fractures in this study probably underestimated the incidence of hip fractures in the real world. Third, this study could not consider socioeconomic factors, such as educational status, income, and marital status, besides indicators of the ability to perform instrumental activities of daily living and participants’ physical functional levels, such as instrumental ADL (IADL), because these factors were not available in the KDB or the dental checkup data of the present study. A previous study reported that tooth loss can have negative impacts on socioeconomic status, self-esteem, and oral health-related quality of life.^42^ The socioeconomic status and the IADL may be confounding factors in this study. Future studies considering these confounding factors are essential. Fourth, it is uncertain whether the findings of the present study are applicable to other countries. This study used data from only one prefecture in Japan, limiting its generalizability. Fifth, the participants included in this study might be healthier than the general older population. Older people with long-term hospitalization and residents at nursing home were not eligible for the public dental checkups in the present study. The participants included in this study had a lower prevalence of LTC certification than those excluded from the study (eTable 1 and eTable 2). The findings of the present study should be verified in the general older population, including those with LTC.

In conclusion, the present large retrospective cohort study of 190,998 older adults showed that the count of tooth loss using sound teeth + filled teeth predicted the risk of hip fractures in women. Our findings suggest that older women with tooth loss are a potential screening target for the prevention of hip fractures in the general population. Local governments should carry out health guidance using a high-risk approach.

## SUPPLEMENTARY MATERIAL

eTable 1. Characteristics of men participants included in and excluded from the present study at baseline checkup

eTable 2. Characteristics of women participants included in and excluded from the present study at baseline checkup

eTable 3. Associations between the number of teeth and the incidence of hip fractures using inverse probability of treatment weighting

eTable 4. Association of number of teeth and incidence of hip fractures during 24-month observational period

eTable 5. Association of number of teeth and incidence of hip fractures during 36-month observational period

eTable 6. Association between the number of sound + filled + decayed teeth in 47,147 men and 62,720 women who use dentures and the incidence of hip fracture

eFigure 1.

## References

[1] Montero-Odasso M, van der Velde N, Martin FC, . World guidelines for falls prevention and management for older adults: a global initiative. Age Ageing. 2022;51(9):afac205. 10.1093/ageing/afac205 36178003 PMC9523684

[2] GBD 2019 Fracture Collaborators. Global, regional, and national burden of bone fractures in 204 countries and territories, 1990–2019: a systematic analysis from the Global Burden of Disease Study 2019. Lancet Healthy Longev. 2021;2(9):e580–e592. 10.1016/S2666-7568(21)00172-0 34723233 PMC8547262

[3] Melton LJ 3rd. Who has osteoporosis? A conflict between clinical and public health perspectives. J Bone Miner Res. 2000;15(12):2309–2314. 10.1359/jbmr.2000.15.12.2309 11127196

[4] Cummings SR, Melton LJ. Epidemiology and outcomes of osteoporotic fractures. Lancet. 2002;359(9319):1761–1767. 10.1016/S0140-6736(02)08657-9 12049882

[5] Kammerlander C, Gosch M, Kammerlander-Knauer U, Luger TJ, Blauth M, Roth T. Long-term functional outcome in geriatric hip fracture patients. Arch Orthop Trauma Surg. 2011;131(10):1435–1444. 10.1007/s00402-011-1313-6 21523326

[6] Xu B, Han L, Liu H, . Cardiovascular disease and hip fracture among older inpatients in Beijing, China. Biomed Res Int. 2013;2013:493696. 10.1155/2013/493696 23936809 PMC3713359

[7] Lloyd R, Baker G, MacDonald J, Thompson NW. Co-morbidities in patients with a hip fracture. Ulster Med J. 2019;88(3):162–166. 31619850 PMC6790636

[8] Malafarina V, Reginster JY, Cabrerizo S, . Nutritional status and nutritional treatment are related to outcomes and mortality in older adults with hip fracture. Nutrients. 2018;10(5). 10.3390/nu10050555 29710860 PMC5986435

[9] Yong EL, Ganesan G, Kramer MS, . Risk factors and trends associated with mortality among adults with hip fracture in Singapore. JAMA Netw Open. 2020;3(2):e1919706. 10.1001/jamanetworkopen.2019.19706 32058551 PMC12124694

[10] Ebeling PR. Hip fractures and aging: a global problem requiring coordinated global solutions. J Bone Miner Res. 2023;38(8):1062–1063. 10.1002/jbmr.4881 37475191

[11] Dyer SM, Crotty M, Fairhall N, . A critical review of the long-term disability outcomes following hip fracture. BMC Geriatr. 2016;16(1):158. 10.1186/s12877-016-0332-0 27590604 PMC5010762

[12] Berry SD, Kiel DP, Colón-Emeric C. Hip fractures in older adults in 2019. JAMA. 2019;321(22):2231–2232. 10.1001/jama.2019.5453 31074763 PMC6800121

[13] Skuladottir SS, Ramel A, Hjaltadottir I, . Characteristics of incidence hip fracture cases in older adults participating in the longitudinal AGES-Reykjavik study. Osteoporos Int. 2021;32(2):243–250. 10.1007/s00198-020-05567-x 32808140 PMC11190885

[14] Yoshida M, Kikutani T, Okada G, Kawamura T, Kimura M, Akagawa Y. The effect of tooth loss on body balance control among community-dwelling elderly persons. Int J Prosthodont. 2009;22(2):136–139. 19418857

[15] Zelig R, Goldstein S, Touger-Decker R, . Tooth loss and nutritional status in older adults: a systematic review and meta-analysis. JDR Clin Trans Res. 2022;7(1):4–15. 10.1177/2380084420981016 33345687

[16] Dantas PP de A, Colussi PRG, Dezingrini K da S, Sachetti DG, Muniz FWMG. Pairs of natural teeth rather than use of dental prosthesis are associated with nutritional status in older adults: a cross-sectional study. J Dent. 2021;108:103656. 10.1016/j.jdent.2021.103656 33819455

[17] Yu YH, Cheung WS, Miller DR, Steffensen B. Number of teeth is associated with hip fracture and femoral neck bone mineral density in the NHANES. Arch Osteoporos. 2021;16(1):105. 10.1007/s11657-021-00970-1 34189624 PMC8312725

[18] Wakai K, Naito M, Naito T, . Tooth loss and risk of hip fracture: a prospective study of male Japanese dentists. Community Dent Oral Epidemiol. 2013;41(1):48–54. 10.1111/j.1600-0528.2012.00706.x 22747907

[19] Priebe J, Wermers RA, Sems SA, Viozzi CF, Koka S. Relationship of number of missing teeth to hip fracture in elderly patients: a cohort pilot study. J Prosthodont. 2019;28(3):258–263. 10.1111/jopr.12626 28913949

[20] Ministry of Health, Labour and Welfare. Manual for Dental Checkups for the older adults in Japan.pdf. Published online 2018. https://www.mhlw.go.jp/content/000410121.pdf; Accessed 1.10.2023.

[21] Khatib R, Santesso N, Pickard L, . Fracture risk in long term care: a systematic review and meta-analysis of prospective observational studies. BMC Geriatr. 2014;14:130. 10.1186/1471-2318-14-130 25471485 PMC4266898

[22] Tamiya N, Noguchi H, Nishi A, . Population ageing and wellbeing: lessons from Japan’s long-term care insurance policy. Lancet. 2011;378(9797):1183–1192. 10.1016/S0140-6736(11)61176-8 21885099

[23] Tsutsui T, Muramatsu N. Care-needs certification in the long-term care insurance system of Japan. J Am Geriatr Soc. 2005;53(3):522–527. 10.1111/j.1532-5415.2005.53175.x 15743300

[24] Brix TH, Lund LC, Henriksen DP, . Methimazole and risk of acute pancreatitis. Lancet Diabetes Endocrinol. 2020;8(3):187–189. 10.1016/S2213-8587(20)30025-5 32035032

[25] Kadesjö E, Roos A, Siddiqui AJ, Sartipy U, Holzmann MJ. Treatment with cardiovascular medications: prognosis in patients with myocardial injury. J Am Heart Assoc. 2021;10(1):e017239. 10.1161/JAHA.120.017239 33372527 PMC7955454

[26] Yoshimura R, Yamamoto R, Otsuki N, . Long-term care needs and incidence of end-stage kidney disease: a retrospective cohort study. J Am Med Dir Assoc. 2023;24(3):402–404. 10.1016/j.jamda.2023.01.008 36764331

[27] Guglielmi V, Bellia A, Pecchioli S, . Effectiveness of adherence to lipid lowering therapy on LDL-cholesterol in patients with very high cardiovascular risk: a real-world evidence study in primary care. Atherosclerosis. 2017;263:36–41. 10.1016/j.atherosclerosis.2017.05.018 28599257

[28] Hayashida K, Murakami G, Matsuda S, Fushimi K. History and profile of diagnosis procedure combination (DPC): development of a real data collection system for acute inpatient care in Japan. J Epidemiol. 2021;31(1):1–11. 10.2188/jea.JE20200288 33012777 PMC7738645

[29] Fine JP, Gray RJ. A proportional hazards model for the subdistribution of a competing risk. J Am Stat Assoc. 1999;94(446):496–509. 10.1080/01621459.1999.10474144

[30] Nishikawa M, Tango T, Ogawa M. Non-parametric inference of adverse events under informative censoring. Stat Med. 2006;25(23):3981–4003. 10.1002/sim.2511 16526008

[31] Austin PC, Fine JP. Practical recommendations for reporting Fine-Gray model analyses for competing risk data. Stat Med. 2017;36(27):4391–4400. 10.1002/sim.7501 28913837 PMC5698744

[32] Chambless LE, Cummiskey CP, Cui G. Several methods to assess improvement in risk prediction models: extension to survival analysis. Stat Med. 2011;30(1):22–38. 10.1002/sim.4026 20827726

[33] Mühlenbruch K, Kuxhaus O, Pencina MJ, Boeing H, Liero H, Schulze MB. A confidence ellipse for the Net Reclassification Improvement. Eur J Epidemiol. 2015;30(4):299–304. 10.1007/s10654-015-0001-1 25724473 PMC4385149

[34] Tanaka R, Tanaka T, Yeung AWK, Taguchi A, Katsumata A, Bornstein MM. Mandibular radiomorphometric indices and tooth loss as predictors for the risk of osteoporosis using panoramic radiographs. Oral Health Prev Dent. 2020;18(4):773–782. 10.3290/j.ohpd.a45081 32895661 PMC11654613

[35] Penoni DC, Torres SR, Oliveira ML, Farias MLF, Vettore MV, Leão ATT. Untreated osteoporosis and higher FRAX as risk factors for tooth loss: a 5-year prospective study. J Bone Miner Metab. 2023;41(5):727–737. 10.1007/s00774-023-01451-w 37432542

[36] Pietschmann P, Rauner M, Sipos W, Kerschan-Schindl K. Osteoporosis: an age-related and gender-specific disease—a mini-review. Gerontology. 2009;55(1):3–12. 10.1159/000166209 18948685

[37] Melton LJ 3rd, Chrischilles EA, Cooper C, Lane AW, Riggs BL. Perspective. How many women have osteoporosis? J Bone Miner Res. 1992;7(9):1005–1010. 10.1002/jbmr.5650070902 1414493

[38] Oie E, Horiuchi M, Soma K. Effects of occlusal contact and its area on gravity fluctuation. Angle Orthod. 2010;80(3):540–546. 10.2319/032309-173.1 20050750 PMC8985731

[39] Yamamoto T, Kondo K, Misawa J, . Dental status and incident falls among older Japanese: a prospective cohort study. BMJ Open. 2012;2(4):e001262. 10.1136/bmjopen-2012-001262 22855628 PMC4400665

[40] McCarthy CJ, Kelly MA, Kenny PJ. Assessment of previous fracture and anti-osteoporotic medication prescription in hip fracture patients. Ir J Med Sci. 2022;191(1):247–252. 10.1007/s11845-021-02571-w 33687665

[41] Loggers SAI, Willems HC, Van Balen R, . Evaluation of quality of life after nonoperative or operative management of proximal femoral fractures in frail institutionalized patients: the FRAIL-HIP Study. JAMA Surg. 2022;157(5):424–434. 10.1001/jamasurg.2022.0089 35234817 PMC8892372

[42] Nordenram G, Davidson T, Gynther G, . Qualitative studies of patients’ perceptions of loss of teeth, the edentulous state and prosthetic rehabilitation: a systematic review with meta-synthesis. Acta Odontol Scand. 2013;71(3–4):937–951. 10.3109/00016357.2012.734421 23101439

