# Supplementary material for: Number of Teeth and Incidence of Hip Fracture in Older Adults Aged ≥75 Years: The OHSAKA Study
Source: J Epidemiol. 2025 Jul 5;35(7):313–20. doi: 10.2188/jea.JE20240165 (PMC12162183; doi:10.2188/jea.JE20240165)
Supplement: Supplementary file 1 [file je-35-313-s001.pdf]

eTable 1. Characteristics of men participants included in and excluded from the present study at baseline checkup

	Included men (n=81,158)	Excluded men (n=12,389)	n (%)
Age, years	79 (77–83)	80 (77–84)	12,389 (100.0)
Long–term care needs levels, n (%)			12,389 (100.0)
None	52,113 (64.2)	6,914 (55.8)	
Requiring support 1	12,542 (15.5)	2,296 (18.5)	
2	5,152 (6.3)	928 (7.5)	
Requiring long–term care 1	5,672 (7.0)	1,094 (8.8)	
2	2,397 (3.0)	504 (4.1)	
3	1,296 (1.6)	247 (2.0)	
4	1,199 (1.5)	225 (1.8)	
5	787 (1.0)	181 (1.5)	
Body mass index, n (%)			8,652 (69.8)
<18.5 kg/m^2	4,665 (5.7)	562 (6.5)	
18.5–24.9 kg/m^2	59,146 (72.9)	6,244 (72.2)	
25.0–29.9 kg/m^2	15,987 (19.7)	1,670 (19.3)	
≥30 kg/m^2	1,360 (1.7)	176 (2.0)	
Smoking, n (%)			12,389 (100.0)
Never	23,734 (29.2)	3,731 (30.1)	
Past	47,871 (59.0)	6,953 (56.1)	
Current	9,553 (11.8)	1,705 (13.8)	
Use of dentures, n (%)	47,147 (58.1)	8,094 (65.3)	12,389 (100.0)
Antidiabetic drugs, n (%)	15,614 (19.2)	2,414 (19.5)	12,389 (100.0)
Antiplatelet drugs, n (%)	24,999 (30.8)	4,031 (32.5)	12,389 (100.0)

Beta-blockers, n (%)	10,998 (13.6)	1,731 (14.0)	12,389 (100.0)
Calcium channel blockers, n (%)	32,268 (39.8)	4,970 (40.1)	12,389 (100.0)
RAS blockers, n (%)	31,659 (39.0)	4,955 (40.0)	12,389 (100.0)
Cholesterol-lowering drugs, n (%)	24,483 (30.2)	3,670 (29.6)	12,389 (100.0)
Antidementia drugs, n (%)	3,006 (3.7)	626 (5.1)	12,389 (100.0)
Osteoporosis drugs, n (%)	3,902 (4.8)	627 (5.1)	12,389 (100.0)
Kidney replacement therapy, n (%)	519 (0.6)	87 (0.7)	12,389 (100.0)
Hospitalization, n (%)	9,709 (12.0)	1,601 (13.0)	12,389 (100.0)

Data are presented as median (interquartile range) or n (%)

eTable 2. Characteristics of women participants included in and excluded from the present study at baseline checkup

	Included women (n=109,840)	Excluded women (n=18,850)	n (%)
Number	109,840	18,850	18,850 (100.0)
Age, years	79 (77–83)	80 (77–84)	18,850 (100.0)
Long–term care needs levels, n (%)			18,850 (100.0)
None	57,793 (52.6)	8,264 (43.8)	
Requiring support 1	26,525 (24.1)	5,134 (27.2)	
2	10,808 (9.8)	2,131 (11.3)	
Requiring long–term care 1	8,614 (7.8)	1,994 (10.6)	
2	2,942 (2.7)	645 (3.4)	
3	1,433 (1.3)	314 (1.7)	
4	1,231 (1.1)	235 (1.2)	
5	494 (0.4)	133 (0.7)	
Body mass index, n (%)			11,247 (59.7)
<18.5 kg/m^2	12,007 (10.9)	1,256 (11.2)	
18.5–24.9 kg/m^2	77,602 (70.7)	7,859 (69.8)	
25.0–29.9 kg/m^2	17,484 (15.9)	1,809 (16.1)	
≥30 kg/m^2	2,747 (2.5)	323 (2.9)	
Smoking, n (%)			18,850 (100.0)
Never	99,121 (90.2)	16,735 (88.8)	
Past	6,562 (6.0)	983 (5.2)	
Current	4157 (3.8)	1,132 (6.0)	
Use of dentures, n (%)	62,720 (57.1)	12,083 (64.1)	18,850 (100.0)
Antidiabetic drugs, n (%)	12,638 (11.5)	2,265 (12.0)	18,850 (100.0)

Antiplatelet drugs, n (%)	26,250 (23.9)	4,839 (25.7)	18,850 (100.0)
Beta-blockers, n (%)	11,231 (10.2)	2,041 (10.8)	18,850 (100.0)
Calcium channel blockers, n (%)	44,285 (40.3)	7,812 (41.4)	18,850 (100.0)
RAS blockers, n (%)	38,874 (35.4)	6,818 (36.2)	18,850 (100.0)
Cholesterol-lowering drugs, n (%)	46,480 (42.3)	7,602 (40.3)	18,850 (100.0)
Antidementia drugs, n (%)	4,807 (4.4)	1,205 (6.4)	18,850 (100.0)
Osteoporosis drugs, n (%)	38,679 (35.2)	6,804 (36.1)	18,850 (100.0)
Kidney replacement therapy, n (%)	275 (0.3)	63 (0.3)	18,850 (100.0)
Hospitalization, n (%)	9,380 (8.5)	1,730 (9.2)	18,850 (100.0)

Data are presented as median (interquartile range) or n (%)

eTable 3. Associations between the number of teeth and the incidence of hip fractures using inverse probability of treatment weighting

	IPTW SHR (95% CI)*		
	S+F+D	S+F	S
Teeth 0 vs. 21–28, n	46,583 men and 57,244 women	42,858 men and 61,311 women	27,068 men and 34,483 women
Men	1.81 (1.18–2.79) 1.00 (reference)	2.30 (1.49–3.54) 1.00 (reference)	4.09 (2.18–7.68) 1.00 (reference)
Women	2.53 (1.78–3.60) 1.00 (reference)	1.99 (1.26–3.15) 1.00 (reference)	1.68 (0.96–2.94) 1.00 (reference)
Teeth 1–5 vs. 21–28, n	47,878 men and 63,543 women	44,369 men and 59,870 women	22,331 men and 32,390 women
Men	1.96 (1.27–3.04) 1.00 (reference)	2.19 (1.50–3.19) 1.00 (reference)	3.16 (1.51–6.61) 1.00 (reference)
Women	2.06 (1.38–3.07) 1.00 (reference)	1.36 (0.86–2.16) 1.00 (reference)	1.33 (0.74–2.41) 1.00 (reference)
Teeth 6–10 vs. 21–28, n	49,756 men and 67,760 women	46,179 men and 63,789 women	19,466 men and 27,476 women
Men	1.46 (0.97–2.20) 1.00 (reference)	1.33 (0.90–1.96) 1.00 (reference)	1.85 (0.98–3.47) 1.00 (reference)
Women	1.66 (1.13–2.44) 1.00 (reference)	1.62 (1.08–2.44) 1.00 (reference)	1.02 (0.59–1.77) 1.00 (reference)
Teeth 11–15 vs. 21–28, n	50,471men and 69,018 women	47,181 men and 65,388 women	30,065 men and 15,503 women
Men	1.39 (0.94–2.06) 1.00 (reference)	1.43 (0.97–2.10) 1.00 (reference)	1.58 (0.93–2.70) 1.00 (reference)
Women	1.44 (1.02–2.03) 1.00 (reference)	1.61 (1.12–2.32) 1.00 (reference)	1.32 (0.84–2.08) 1.00 (reference)
Teeth 16–20 vs. 21–28, n	54,258 men and 74,804 women	51,223 men and 71,385 women	9.783 men and 7,828 women
Men	1.21 (0.84–1.75) 1.00 (reference)	1.37 (0.98–1.92) 1.00 (reference)	1.36 (0.71–2.63) 1.00 (reference)
Women	1.32 (0.95–1.82) 1.00 (reference)	1.43 (1.04–1.97) 1.00 (reference)	1.12 (0.75–1.67) 1.00 (reference)

CI, confidence interval; IPTW, inverse probability of treatment weighting; SHR, sub-distribution hazard ratio; S+F+D, sound teeth + filled teeth + decayed teeth; S+F, sound teeth + filled teeth; S, sound teeth.

*Adjusting for age (years), long-term care needs level, body mass index (kg/m^2; <18.5, 18.5–24.9, 25.0–29.9, ≥30), smoking (never, past, current), use of antidiabetic drugs, antiplatelet drugs, beta-blockers, calcium channel blockers, RAS blockers, cholesterol-lowering drugs, antidementia drugs, and osteoporosis drugs, kidney replacement therapy (hemodialysis, peritoneal dialysis, and kidney

transplantation), hospitalization, and 6-month medical costs (<10,000, 10,000–49,999, 50,000–99,999, 100,000–199,999, 200,000–299,999, ≥300,000 yen)

eTable 4. Association of number of teeth and incidence of hip fractures during 24-month observational period

		Number of sound + filled + decayed teeth					
		0	1–5	6–10	11–15	16–20	21–28
Men	Observational period, months	24 (24–24)	24 (24–24)	24 (24–24)	24 (24–24)	24 (24–24)	24 (24–24)
	Incidence of fracture, n (%)	51 (1.1)	55 (0.9)	54 (0.7)	51 (0.6)	72 (0.6)	175 (0.4)
	All-cause mortality, n (%)	506 (10.9)	488 (8.2)	582 (7.5)	568 (6.7)	681 (5.5)	1,697 (4.0)
	Unadjusted SHR (95% CI)	2.40 (1.69–3.41)	2.17 (1.55–3.03)	1.45 (1.03–2.04)	1.44 (1.07–1.95)	1.40 (1.02–1.92)	1.00 (reference)
	Adjusted SHR (95% CI)*	1.02 (0.54–1.92)	1.04 (0.63–1.72)	0.87 (0.54–1.40)	0.78 (0.55–1.12)	1.05 (0.67–1.65)	1.00 (reference)
Women	Observational period, months	24 (24–24)	24 (24–24)	24 (24–24)	24 (24–24)	24 (24–24)	24 (24–24)
	Incidence of fracture, n (%)	133 (2.9)	127 (1.8)	196 (1.8)	199 (1.6)	274 (1.5)	557 (1.0)
	All-cause mortality, n (%)	294 (6.3)	292 (4.2)	385 (3.5)	393 (3.2)	409 (2.3)	1,027 (1.8)
	Unadjusted SHR (95% CI)	2.93 (2.39–3.58)	1.95 (1.54–2.47)	1.76 (1.48–2.08)	1.62 (1.33–1.97)	1.51 (1.31–1.74)	1.00 (reference)
	Adjusted SHR (95% CI)*	1.42 (1.12–1.79)	1.21 (0.93–1.57)	1.16 (0.94–1.42)	1.19 (0.97–1.46)	1.41 (1.22–1.64)	1.00 (reference)

CI, confidence interval; SHR, sub-distribution hazard ratio.

*Adjusting for age (years), long-term care needs level, body mass index (kg/m^2; <18.5, 18.5–24.9, 25.0–29.9, ≥30), smoking (never, past, current), use of antidiabetic drugs, antiplatelet drugs, beta-blockers, calcium channel blockers, RAS blockers, cholesterol-lowering drugs, antidementia drugs, and osteoporosis drugs, kidney replacement therapy (hemodialysis, peritoneal dialysis, and kidney transplantation), hospitalization, and 6-month medical costs (<10,000, 10,000–49,999, 50,000–99,999, 100,000–199,999, 200,000–299,999, ≥300,000 yen)

eTable 5. Association of number of teeth and incidence of hip fractures during 36-month observational period

		Number of sound + filled + decayed teeth					
		0	1–5	6–10	11–15	16–20	21–28
Men	Observational period, months	36 (28–36)	36 (30–36)	36 (31–36)	36 (32–36)	36 (32–36)	36 (32–36)
	Incidence of fracture, n (%)	78 (1.7)	73 (1.2)	73 (0.9)	92 (1.1)	105 (0.9)	266 (0.6)
	All-cause mortality, n (%)	921 (19.9)	971 (16.4)	1,202 (15.4)	1,212 (14.2)	1,429 (11.6)	3,718 (8.9)
	Unadjusted SHR (95% CI)	2.97 (2.29–3.85)	2.14 (1.60–2.87)	1.65 (1.23–2.20)	1.63 (1.27–2.09)	1.34 (1.05–1.73)	1.00 (reference)
	Adjusted SHR (95% CI)*	1.47 (1.03–2.09)	1.07 (0.75–1.53)	1.14 (0.79–1.64)	1.08 (0.77–1.51)	1.01 (0.72–1.42)	1.00 (reference)
Women	Observational period, months	36 (30–36)	36 (32–36)	36 (32–36)	36 (32–36)	36 (32–36)	36 (32–36)
	Incidence of fracture, n (%)	199 (4.3)	202 (2.9)	309 (2.8)	304 (2.5)	412 (2.3)	885 (1.6)
	All-cause mortality, n (%)	548 (11.8)	594 (8.6)	795 (7.2)	762 (6.2)	949 (5.2)	2,264 (4.0)
	Unadjusted SHR (95% CI)	3.16 (2.59–3.86)	1.98 (1.65–2.37)	1.86 (1.59–2.17)	1.63 (1.43–1.86)	1.50 (1.35–1.67)	1.00 (reference)
	Adjusted SHR (95% CI)*	1.36 (1.11–1.66)	1.07 (0.87–1.32)	1.21 (1.00–1.46)	1.12 (0.95–1.32)	1.28 (1.12–1.46)	1.00 (reference)

CI, confidence interval; SHR, sub-distribution hazard ratio.

*Adjusting for age (years), long-term care needs level, body mass index (kg/m^2; <18.5, 18.5–24.9, 25.0–29.9, ≥30), smoking (never, past, current), use of antidiabetic drugs, antiplatelet drugs, beta-blockers, calcium channel blockers, RAS blockers, cholesterol-lowering drugs, antidementia drugs, and osteoporosis drugs, kidney replacement therapy (hemodialysis, peritoneal dialysis, and kidney transplantation), hospitalization, and 6-month medical costs (<10,000, 10,000–49,999, 50,000–99,999, 100,000–199,999, 200,000–299,999, ≥300,000 yen)

eTable 6. Association between the number of sound + filled + decayed teeth in 47,147 men and 62,720 women who use dentures and the incidence of hip fracture

		Number of sound + filled + decayed teeth					
		0	1–5	6–10	11–15	16–20	21–28
Men	Observational period, months	36 (27–43)	37 (29–44)	38 (30–44)	39 (32–44)	40 (32–44)	40 (32–44)
	Incidence of fracture, n (%)	108 (2.4)	118 (2.1)	111 (1.5)	114 (1.4)	136 (1.4)	124 (1.1)
	All-cause mortality, n (%)	887 (19.6)	914 (15.9)	1146 (15.3)	1099 (14.0)	1094 (11.0)	1064 (9.2)
	Unadjusted SHR (95% CI)	2.25 (1.72–2.95)	1.91 (1.50–2.43)	1.34 (1.01–1.79)	1.32 (1.02–1.72)	1.27 (0.99–1.62)	1.00 (reference)
	Adjusted SHR (95% CI)*	1.06 (0.76–1.49)	1.15 (0.87–1.53)	0.85 (0.63–1.16)	0.93 (0.68–1.25)	1.00 (0.73–1.37)	1.00 (reference)
Women	Observational period, months	36 (29–43)	37 (30–44)	39 (32–44)	39 (32–44)	40 (32–44)	40 (32–44)
	Incidence of fracture, n (%)	277 (6.1)	315 (4.7)	442 (4.1)	434 (3.8)	459 (3.2)	362 (2.4)
	All-cause mortality, n (%)	519 (11.5)	551 (8.2)	726 (6.8)	668 (5.8)	712 (4.9)	592 (4.0)
	Unadjusted SHR (95% CI)	2.65 (2.29–3.06)	1.98 (1.72–2.29)	1.72 (1.50–1.97)	1.55 (1.37–1.76)	1.31 (1.17–1.48)	1.00 (reference)
	Adjusted SHR (95% CI)*	1.36 (1.16–1.61)	1.25 (1.06–1.47)	1.20 (1.03–1.40)	1.26 (1.09–1.45)	1.23 (1.07–1.41)	1.00 (reference)

CI, confidence interval; SHR, sub-distribution hazard ratio.

*Adjusting for age (years), long-term care needs level, body mass index (kg/m^2; <18.5, 18.5–24.9, 25.0–29.9, ≥30), smoking (never, past, current), use of antidiabetic drugs, antiplatelet drugs, beta-blockers, calcium channel blockers, RAS blockers, cholesterol-lowering drugs, antidementia drugs, and osteoporosis drugs, kidney replacement therapy (hemodialysis, peritoneal dialysis, and kidney transplantation), hospitalization, and 6-month medical costs (<10,000, 10,000–49,999, 50,000–99,999, 100,000–199,999, 200,000–299,999, ≥300,000 yen)

eFigure 1

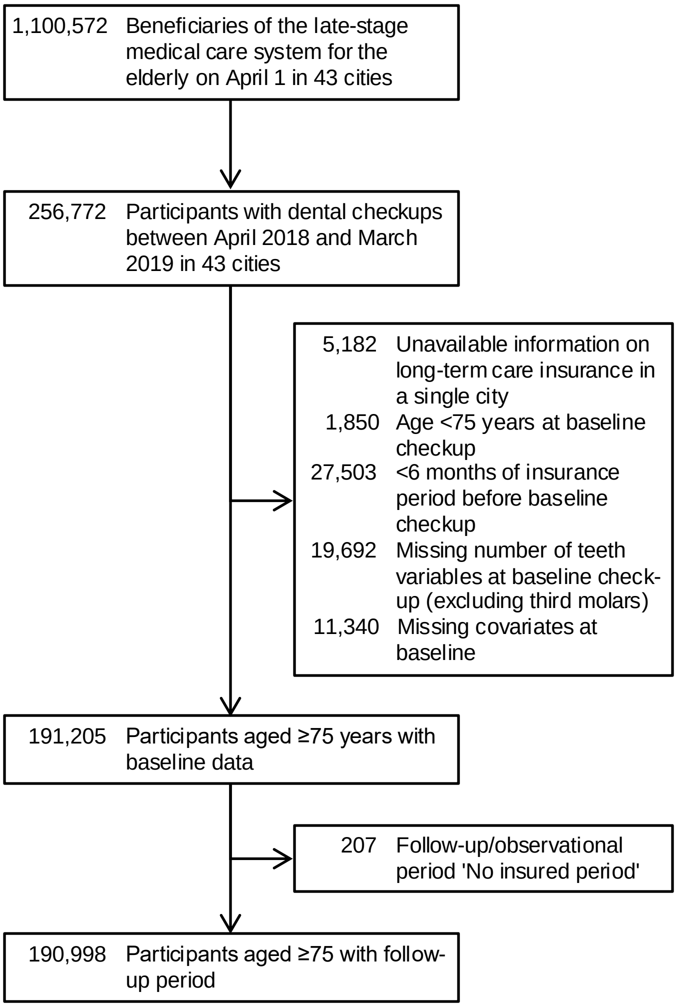
1,100,572 Beneficiaries of the late-stage medical care system for the elderly on April 1 in 43 cities
256,772 Participants with dental checkups between April 2018 and March 2019 in 43 cities
5,182 Unavailable information on long-term care insurance in a single city
1,850 Age <75 years at baseline checkup
27,503 <6 months of insurance period before baseline checkup
19,692 Missing number of teeth variables at baseline check-up (excluding third molars)
11,340 Missing covariates at baseline
191,205 Participants aged ≥75 years with baseline data
207 Follow-up/observational period 'No insured period'
190,998 Participants aged ≥75 with follow-up period
